# Pan-cancer immunogenic death analysis identifies key roles of CXCR3 and CCL18 in hepatocellular carcinoma

**DOI:** 10.1016/j.gendis.2023.04.007

**Published:** 2023-05-10

**Authors:** Jia Guo, Xin Sun, Guowei Pan, Ce Li, Xin He, Xiaofang Che, Zan Teng, Xiujuan Qu, Yunpeng Liu, Bowen Yang

**Affiliations:** a Department of Medical Oncology, Provincial Key Laboratory of Anticancer Drugs and Biotherapy of Liaoning Province, Liaoning Provincial Clinical Research Center for Cancer, Clinical Cancer Research Center of Shenyang, The First Hospital of China Medical University, Shenyang, Liaoning 110001, China; b Research Center for Universal Health, School of Public Health, China Medical University, Shenyang, Liaoning 110122, China

Immunogenic death is a form of programmed cell death that is common in the development and progression of cancer. However, its impact on tumor progression differs depending on the location of the tumor. Meanwhile, it also plays an important role in antitumor immunity because the process of immunogenic death can release substances that activate immunity.^1^ Therefore, in this study, the key mechanism of mediating immunogenic death in hepatocellular carcinoma (HCC) was found from the excavation of the immunogenic death characteristics of pan-cancer and the in-depth investigation of its effects on different tumor types (Fig. 1A)

**Figure 1:**
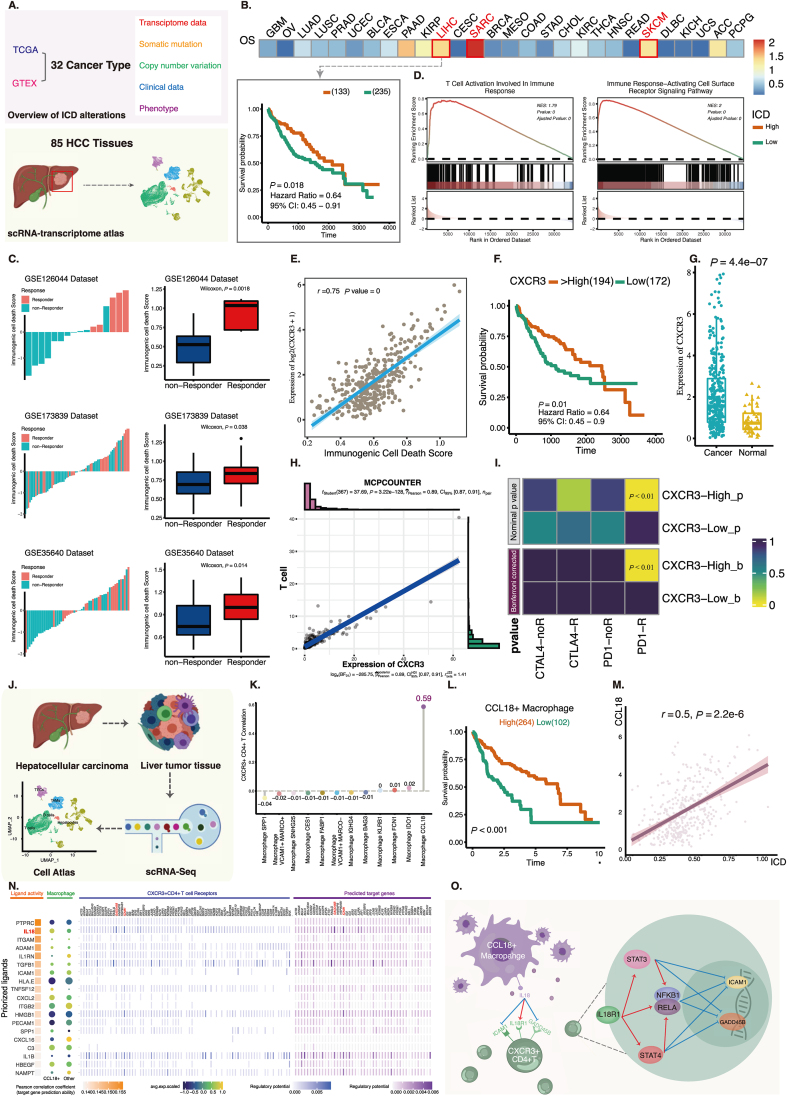
The interaction between CXCR3^+^ T cells and CCL18^+^ macrophages plays an essential role in the process of immunogenic cell death of hepatocellular carcinoma. **(A)** Workflow chart. **(B)** Kaplan–Meier analysis of overall survival according to the ICD score among cancers. **(C)** Gene set enrichment analysis of ICD_high_ tumors in TCGA-LIHC. **(D)** ICB response of patients in three independent immunotherapy datasets. Patients were grouped according to their ICD scores. **(E)** Correlation of ICD score with the expression level of CXCR3 in TCGA-LIHC. **(F)** Kaplan–Meier analysis shows the overall survival in TCGA-LIHC. **(G)** The differential expression level of CXCR3 in HCC and normal liver tissue. **(H)** Correlation between CXCR3 expression and T-cell infiltration levels in hepatocellular carcinoma. **(I)** Immunotherapy efficacy prediction of TCGA-LIHC dataset. **(J)** Data collection and collation. **(K)** Correlations of distinct macrophage subsets with CXCR3^+^ Treg in their cellular proportion. **(L)** Survival analysis of CCL18^+^ macrophages in TCGA-LIHC data performed by CibersortX. **(M)** The scatterplots showing correlations of ICD score with the expression level of CCL18 in TCGA LIHC data. **(N)** NicheNet analysis reveals the ligand activities and ligand expression level based on the key signature genes of CCL18^+^ macrophages as well as receptors and predicted target genes. **(O)** Possible pathways inferred by NicheNet analysis.

Considering the importance of cell death in tumor development and treatment, we first characterized immunogenic death in the pan-cancer landscape. Similar to previous reports, the genomic alterations and expression trends of genes associated with immunogenic death differed a lot in different tumor types (Fig. S1, 2). It points out that the use of a uniform standard to measure the characteristics of pan-cancer is crude and inaccurate. Also, the impact of immunogenic cell death on prognosis varies depending on the type of cancer (Fig. 1B). In HCC, patients with high immunogenic death scores achieved significant benefits in OS and PFS, reflecting a similar profile to highly immunogenic tumors such as melanoma. Also, high levels of immunogenic death at baseline could predict significant immunotherapy efficacy (Fig. 1C). Patients with high ICD levels who survived well had significant enrichment of related signaling pathways such as T-cell activation (Fig. 1D). Therefore, we focused our next analysis on HCC (Fig. S3).

Among the many immunogenic death-related genes, CXCR3 shows a high correlation with immunogenic death score (Fig. 1E), as well as immune-related genes such as CD8A and CD4, demonstrating that CXCR3 plays a central function in immunogenic death in HCC. As expected, CXCR3 levels also distinguished patients' survival outcomes (Fig. 1F), and the levels of CXCR3 differed significantly in cancer and adjacent normal tissues (Fig. 1G). In HCC, CXCR3 expression showed a significant positive correlation with T cells infiltration (Fig. 1H), and further analysis suggested that HCC patients with high CXCR3 expression levels tended to benefit from immunotherapy, further confirming the importance of CXCR3 in HCC treatment (Fig. 1I). Indeed, CXCR3 has been mentioned as a response marker for immunotherapy in recent years but has not been reported in immunogenic death in HCC.

By single-cell transcriptome analysis, we classified cells into five clusters, in which T-cell and macrophage subpopulations showed high immunogenic death characteristics (Fig. 1J; Fig. S4). Single-cell transcriptome analysis suggested that CXCR3 was mainly expressed on the surface of CD4^+^ T cells, so we identified CXCR3^+^ CD4^+^ T cells, a cluster of CD4^+^ T cells in an exhaustion state (Fig. S5, 6). Previous literature confirms a direct interaction between T cells and macrophages, and we found an obvious correlation of CCL18^+^ macrophages with the proportion of CXCR3^+^ CD4^+^ T cells in the immune microenvironment of HCC (Fig. 1K), demonstrating a potential reciprocal relationship. High levels of CCL18^+^ macrophages suggest a better prognosis for HCC patients (Fig. 1L), and CCL18 showed a significant positive correlation with the levels of immunogenic death (Fig. 1M), further confirming the importance of the reciprocal connection between CXCR3^+^ CD4^+^ T cells and CCL18^+^ macrophages during the process of immunogenic death. Finally, we predicted the direct link between the two cells by cell–cell communication. Compared with other macrophages, CCL18^+^ macrophages significantly overexpressed IL18-related genes and interacted with the CD4^+^ T cell surface receptor, GADD45B and ICAM1 (Fig. 1N). On the left side of Figure 1N, CCL18^+^ macrophages significantly overexpressed IL18 ligands compared with other macrophages, while among the receptors for IL18, GADD45B and ICAM1 were labeled as the most active receptors on the surface of CXCR3^+^ CD4^+^ T cells. At the same time, IL18 could also regulate the expression of GADD45B and ICAM1 as target genes. That is, IL18 acts both by acting directly on cell surface receptors and by regulating the transcription of target genes. Thus, based on previous studies reporting on the downstream pathways of IL18 and its receptors, in conjunction with NicheNet's speculation, we hypothesized that CCL18^+^ macrophages secrete IL18 and act on CXCR3^+^ CD4^+^ T cell surface receptors to inhibit the downstream pathways of GADD45B and ICAM1 directly or indirectly, dismantling their pro-tumor effects (Fig. 1O).^2^^,^^3^ In pan-cancer single-cell transcriptome analysis, we found a positive correlation between these two kinds of immune cells and ICD scores, suggesting that this correlation may be a pan-cancer character (Fig. S7).

Our work sheds light on the importance of CXCR3 and CCL18 as the essential markers of immunogenic cell death in HCC, which could be served as biomarkers of prognosis and immunotherapy efficacy in the future. Although previous studies have confirmed that CCL18^+^ macrophages exhibit a pro-tumor immunosuppressive profile in breast and colorectal cancers by secreting CCL18, while in the present study, CCL18^+^ macrophages overexpressed IL18 and played a positive role in immunogenic death in HCC which is supported by its capacity of survival prediction.^4^^,^^5^ Thus, our study suggests that the role of CCL18^+^ macrophages in HCC may be complex, and further research needs to be conducted.

## Conflict of interests

The authors declare that they have no known competing financial interests or personal relationships that could have appeared to influence the work reported in this paper.
