# Supplementary material for: Pan-cancer immunogenic death analysis identifies key roles of CXCR3 and CCL18 in hepatocellular carcinoma
Source: Genes Dis. 2023 May 10;11(2):568–70. doi: 10.1016/j.gendis.2023.04.007 (PMC10491906; doi:10.1016/j.gendis.2023.04.007)
Supplement: Multimedia component 1 [file mmc1.docx]

# Materials and Methods

## Data downloading and processing

For the development of immunogenic cell death signature (ICDs) and the comparison between normal tissues and malignant superfluous tissues, transcriptomic data of The Cancer Genome Atlas (TCGA) pan-cancer cohort and the Genotype-tissue expression (GTEx) cohort were downloaded from the UCSC Xena data portal (https://xenabrowser.net)[1]. Similarly, we systematically collected mutation data, copy number variation, clinical information as well as phenotypes from the same website.

To investigate the predictive function of ICD in immunotherapy, GSE126044, GSE173839, and GSE35640 were analyzed separately[2-4]. A total of 85 tissues with single-cell RNA sequencing results who suffered from hepatocellular carcinoma (HCC) from GSE151530, GSE149614, and GSE156625 were included for further analysis[5-7].

## Evaluation of Immunogenic Cell Death Score and Immune Infiltration

The ICD score was calculated based on the single-sample Gene-Set Enrichment Analysis (ssGSEA) using the immunogenic-cell-death gene set (Supplementary Table 1) collected from the previous article to quantify the expression levels of these genes for each independent tissue[8]. We compared the ICD score between tumor and normal samples in 26 cancers from the TCGA and matched normal tissues from GTEx and the relative change of ICD-related genes.

R package “IOBR” was used to evaluate the relative fraction of immune cell subtypes[9]. Cibersort analysis was used to compare the correlations between the 22 immune cell types and ICD scores in diverse cancers and MCPCOUNTER and QUANTISEQ were utilized for seeking the most essential immune cell types correlated with hub gene[10].

## Survival analysis

We compared the overall survival (OS) and progression-free interval (PFI) of patients from separated cancer types. Kaplan–Meier (K-M) curves were carried out to compare the survival time differences. The association between the ICD score and survival outcomes were analyzed by Cox proportional hazards regression analysis. P-values from log-rank tests were calculated, and less than 0.05 was considered statistically significant.

## Gene set enrichment analysis

To explore the potential biological functions of the ICD-related gene set between low and high groups in each cancer, we conducted Gene Set Enrichment Analysis (GSEA) based on the curated gene sets c2.cp.kegg. v7.0. symbols[11]. We took hepatocellular carcinoma as an example. Normalized P-value <0.05 was regarded as statistical significance.

## Dimension reduction and clustering analysis

By using the R package Seurat v3[12], we performed principal component analysis (PCA) and used FindCluster to visualize cells with the uniform manifold approximation and projection (UMAP) algorithm. We performed a harmony algorithm in the Harmony R package to eliminate the batch effect before clustering. Cells were clustered twice, for the first time they were partitioned into hepatocytes, tumor-associated endothelial cells (TEC), tumor-associated macrophage (TAM), T, and B cells in the first stage. And specific cell types, i.e., T cell and tumor-associated macrophage, were clustered from multiple samples into distinct subtypes in the second stage. Finally, we defined 32 T cell types and 12 macrophage subtypes in HCC based on the gene signatures of each cell type and known lineage markers.

## Trajectory reconstruction based on RNA velocity estimation

To investigate the origin of differentiation for T cells and macrophages, we analyzed expression dynamics by estimating the RNA velocities of single cells. We used the R package ‘Monocle3’ to calculate the RNA velocity value of each gene in each cell and embed the RNA velocity vector in a low-dimensional space and then visualized it on the UMAP projection using Gaussian smoothing on a regular grid[13].

## Transcription factor regulon analysis

The analysis of the regulatory network and regulon activity was performed by SCENIC. The regulon activity was analyzed by the AUCell module of the SCENIC, and the active regulons were determined by the AUCell default threshold[14].

## Characterization of cell-type infiltration based on single-cell expression matrix

To establish the proportions of our defined subdivided cell types from bulk RNA-seq, we used the online tool CIBERSORTx to create a reference signature matrix from our single-cell RNA-seq dataset and estimate cell-type proportions from TCGA-LIHC dataset[15]. Spearman’s correlation analysis was performed to assess the relationship among the proportions of cell-type infiltration.

## Cell-cell interaction analysis between key cell subtypes

Nichenet analysis was conducted for seeding the potential interaction between CXCR3+ Treg and CCL18+ macrophage[16]. CD4+ memory T cells were selected to serve as the reference cell groups.

## Statistical analysis

R software (version 4.1.0) (https://www.r-project.org/) was used in this analysis. Differences between the two groups was analyzed using the Wilcoxon’s test. The differences in OS and PFI between groups were determined by Kaplan-Meier analysis and Cox proportional hazards regression analysis. Results were considered statistically significant when the p < 0.05.

# Reference

1. Goldman, M.J., et al., *Visualizing and interpreting cancer genomics data via the Xena platform.* Nat Biotechnol, 2020. **38**(6): p. 675-678.

2. Cho, J.W., et al., *Genome-wide identification of differentially methylated promoters and enhancers associated with response to anti-PD-1 therapy in non-small cell lung cancer.* Exp Mol Med, 2020. **52**(9): p. 1550-1563.

3. Pusztai, L., et al., *Durvalumab with olaparib and paclitaxel for high-risk HER2-negative stage II/III breast cancer: Results from the adaptively randomized I-SPY2 trial.* Cancer Cell, 2021. **39**(7): p. 989-998 e5.

4. Ulloa-Montoya, F., et al., *Predictive gene signature in MAGE-A3 antigen-specific cancer immunotherapy.* J Clin Oncol, 2013. **31**(19): p. 2388-95.

5. Ma, L., et al., *Single-cell atlas of tumor cell evolution in response to therapy in hepatocellular carcinoma and intrahepatic cholangiocarcinoma.* J Hepatol, 2021. **75**(6): p. 1397-1408.

6. Lu, Y., et al., *A single-cell atlas of the multicellular ecosystem of primary and metastatic hepatocellular carcinoma.* Nat Commun, 2022. **13**(1): p. 4594.

7. Sharma, A., et al., *Onco-fetal Reprogramming of Endothelial Cells Drives Immunosuppressive Macrophages in Hepatocellular Carcinoma.* Cell, 2020. **183**(2): p. 377-394 e21.

8. Hanzelmann, S., R. Castelo, and J. Guinney, *GSVA: gene set variation analysis for microarray and RNA-seq data.* BMC Bioinformatics, 2013. **14**: p. 7.

9. Zeng, D., et al., *IOBR: Multi-Omics Immuno-Oncology Biological Research to Decode Tumor Microenvironment and Signatures.* Front Immunol, 2021. **12**: p. 687975.

10. Chen, B., et al., *Profiling Tumor Infiltrating Immune Cells with CIBERSORT.* Methods Mol Biol, 2018. **1711**: p. 243-259.

11. Kanehisa, M., et al., *KEGG: new perspectives on genomes, pathways, diseases and drugs.* Nucleic Acids Res, 2017. **45**(D1): p. D353-D361.

12. Stuart, T., et al., *Comprehensive Integration of Single-Cell Data.* Cell, 2019. **177**(7): p. 1888-1902 e21.

13. Cao, J., et al., *The single-cell transcriptional landscape of mammalian organogenesis.* Nature, 2019. **566**(7745): p. 496-502.

14. Aibar, S., et al., *SCENIC: single-cell regulatory network inference and clustering.* Nat Methods, 2017. **14**(11): p. 1083-1086.

15. Newman, A.M., et al., *Determining cell type abundance and expression from bulk tissues with digital cytometry.* Nat Biotechnol, 2019. **37**(7): p. 773-782.

16. Browaeys, R., W. Saelens, and Y. Saeys, *NicheNet: modeling intercellular communication by linking ligands to target genes.* Nat Methods, 2020. **17**(2): p. 159-162.
