# Supplementary material for: Pan-cancer immunogenic death analysis identifies key roles of CXCR3 and CCL18 in hepatocellular carcinoma
Source: Genes Dis. 2023 May 10;11(2):568–70. doi: 10.1016/j.gendis.2023.04.007 (PMC10491906; doi:10.1016/j.gendis.2023.04.007)
Supplement: Multimedia component 2 [file mmc2.docx]

Supplementary Material

# Supplementary Table 1. Immunogenic-cell-death related genes

| **Gene Name** | **Official Full Name** | **Chromosome** |
| --- | --- | --- |
| ATG5 | autophagy related 5 | 6 |
| BAX | BCL2 associated X, apoptosis regulator | 19 |
| CALR | calreticulin | 19 |
| CASP1 | caspase 1 | 11 |
| CASP8 | caspase 8 | 2 |
| CD4 | CD4 molecule | 12 |
| CD8A | CD8a molecule | 2 |
| CD8B | CD8b molecule | 2 |
| CXCR3 | C-X-C motif chemokine receptor 3 | X |
| EIF2AK3 | eukaryotic translation initiation factor 2 alpha kinase 3 | 2 |
| ENTPD1 | ectonucleoside triphosphate diphosphohydrolase 1 | 10 |
| FOXP3 | forkhead box P3 | X |
| HMGB1 | high mobility group box 1 | 13 |
| HSP90AA1 | heat shock protein 90 alpha family class A member 1 | 14 |
| IFNA1 | interferon alpha 1 | 9 |
| IFNB1 | interferon beta 1 | 9 |
| IFNG | interferon gamma | 12 |
| IFNGR1 | interferon gamma receptor 1 | 6 |
| IL10 | interleukin 10 | 1 |
| IL17A | interleukin 17A | 6 |
| IL17RA | interleukin 17 receptor A | 22 |
| IL1B | interleukin 1 beta | 2 |
| IL1R1 | interleukin 1 receptor type 1 | 2 |
| IL6 | interleukin 6 | 7 |
| LY96 | lymphocyte antigen 96 | 8 |
| MYD88 | MYD88 innate immune signal transduction adaptor | 3 |
| NLRP3 | NLR family pyrin domain containing 3 | 1 |
| NT5E | 5'-nucleotidase ecto | 6 |
| P2RX7 | purinergic receptor P2X 7 | 12 |
| PDIA3 | protein disulfide isomerase family A member 3 | 15 |
| PIK3CA | phosphatidylinositol-4,5-bisphosphate 3-kinase catalytic subunit alpha | 3 |
| PRF1 | perforin 1 | 10 |
| TLR4 | toll like receptor 4 | 9 |
| TNF | tumor necrosis factor | 6 |
