# Supplementary figures and images for: Pan-cancer immunogenic death analysis identifies key roles of CXCR3 and CCL18 in hepatocellular carcinoma
Source: Genes Dis. 2023 May 10;11(2):568–70. doi: 10.1016/j.gendis.2023.04.007 (PMC10491906; doi:10.1016/j.gendis.2023.04.007)

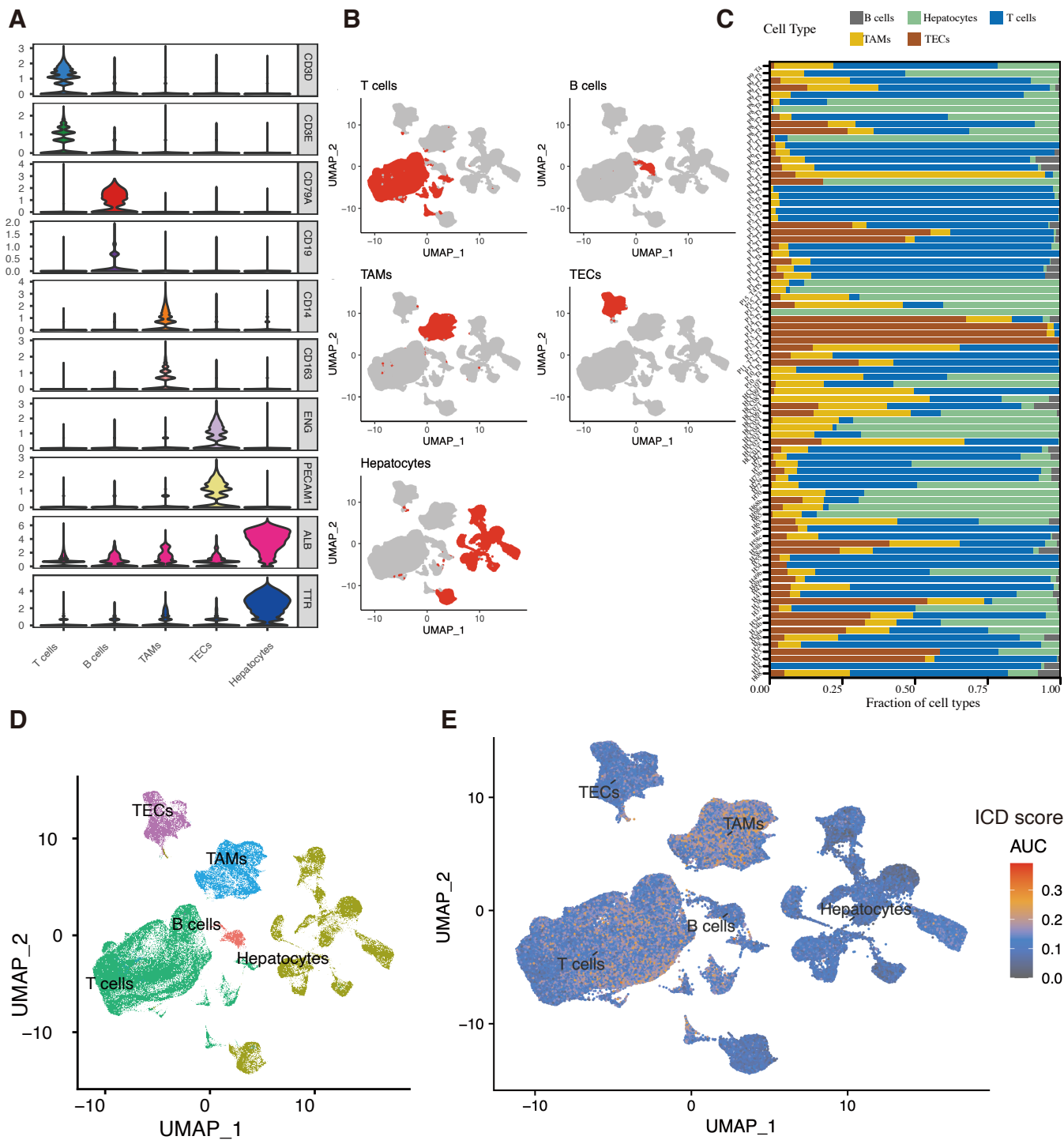

Supplement: Multimedia component 3 [file mmc3.pdf]

A.

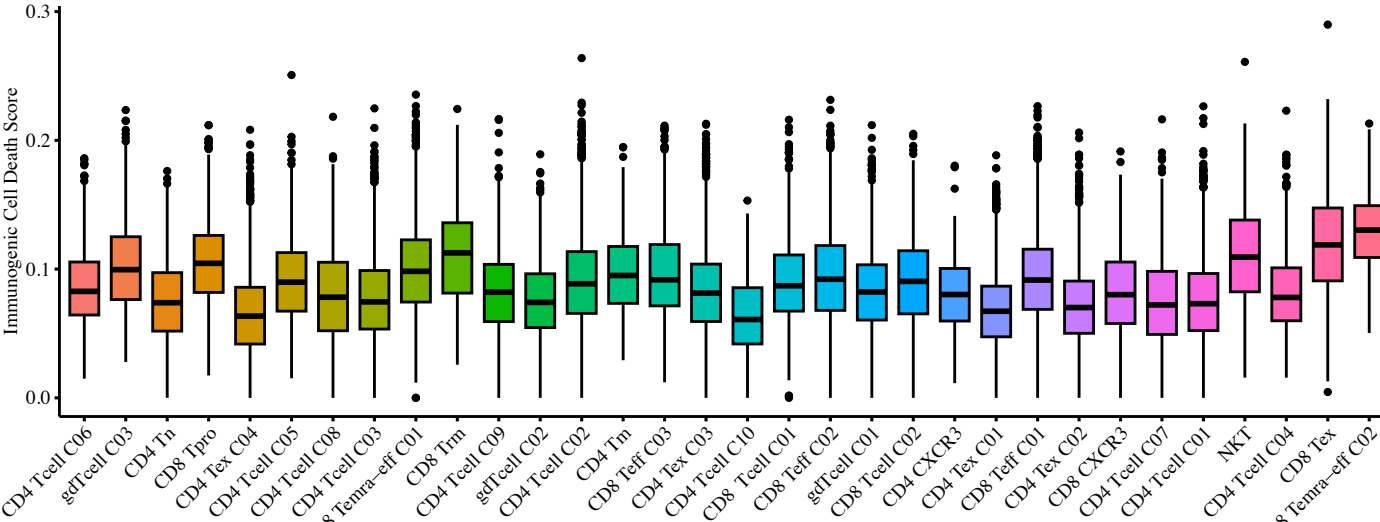

**B.**

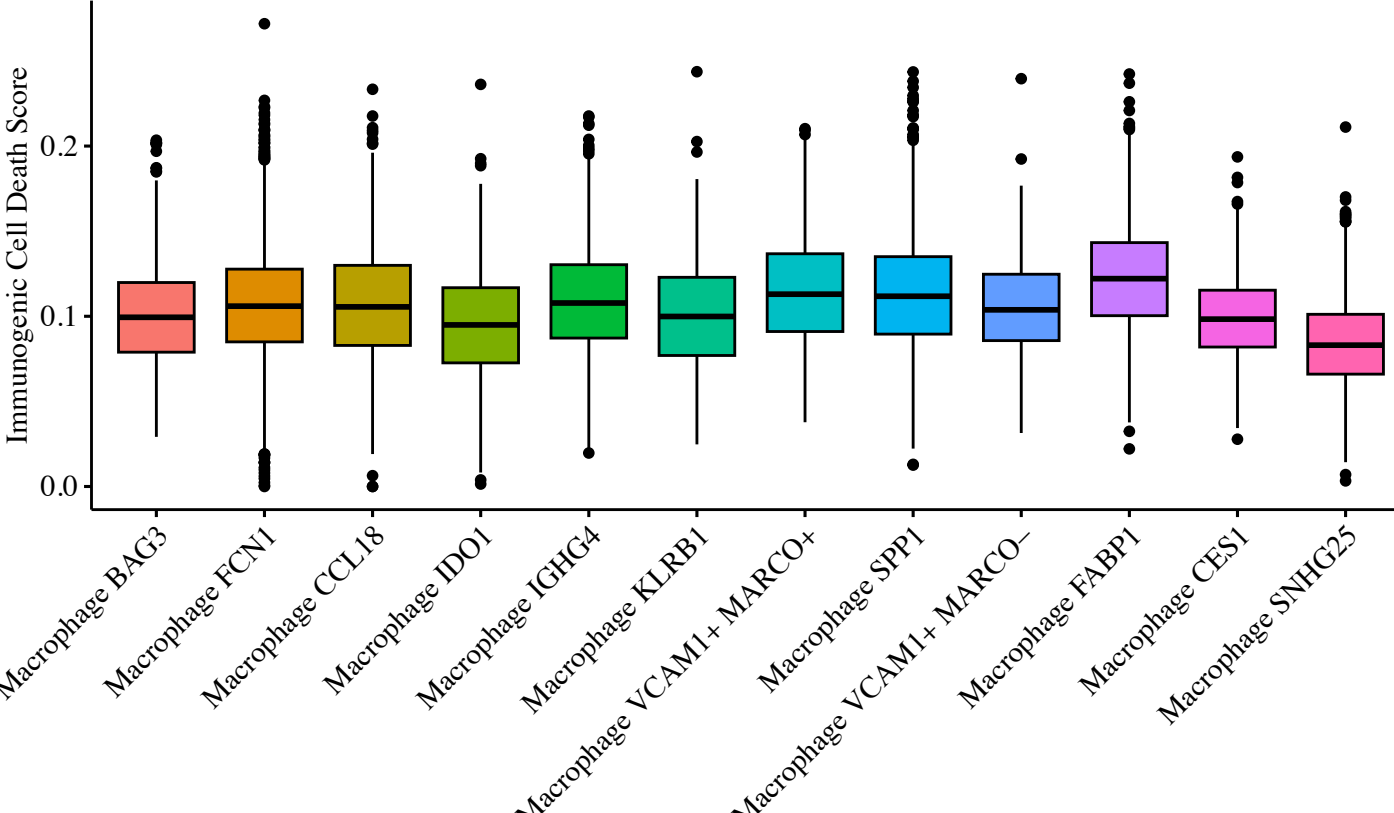

Supplement: Multimedia component 4 [file mmc4.pdf]

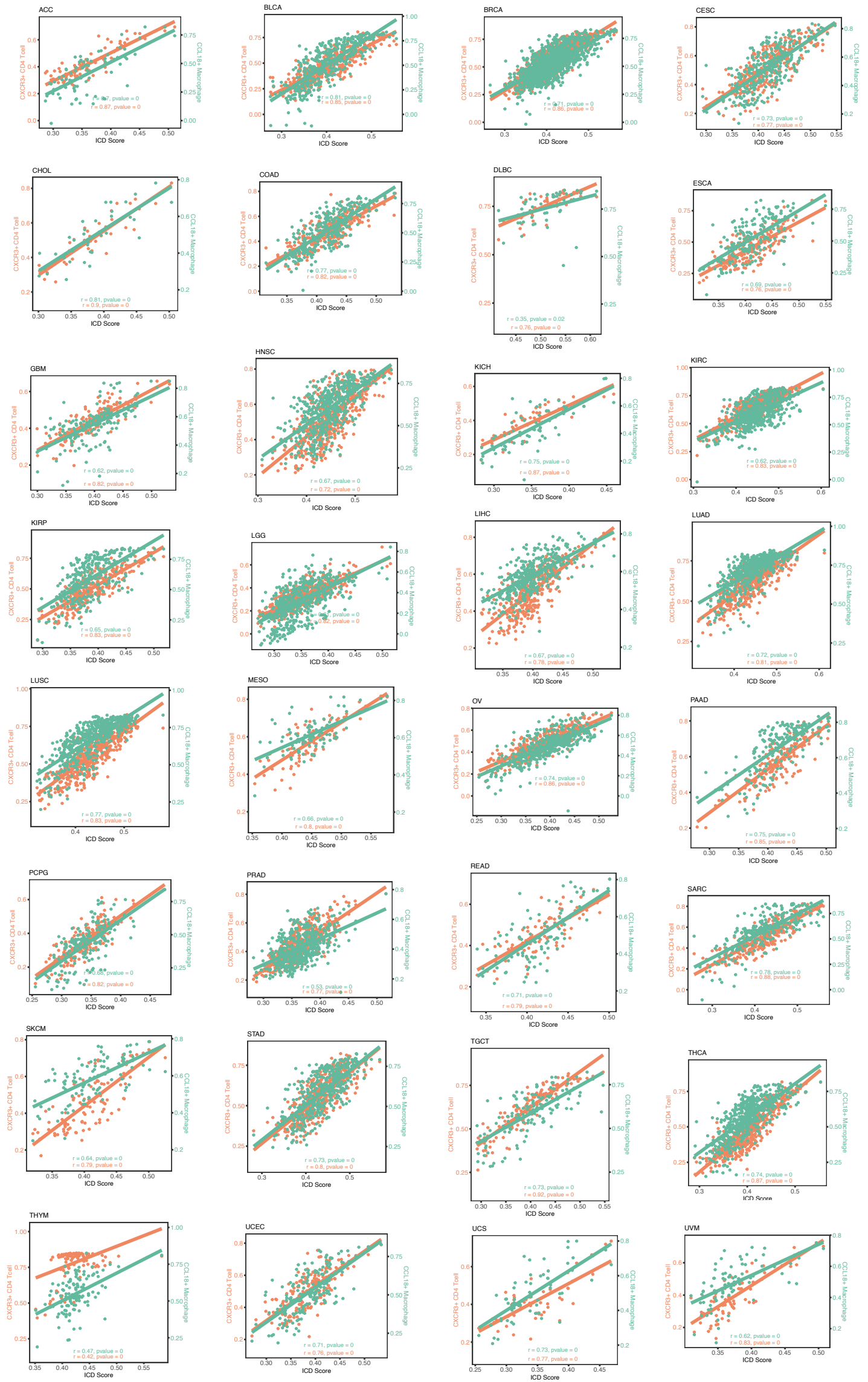

Supplement: Multimedia component 5 [file mmc5.pdf]

**A**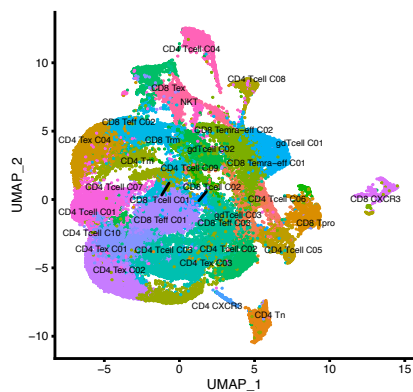**B**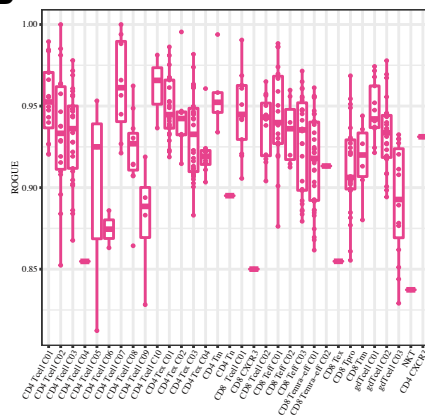**C**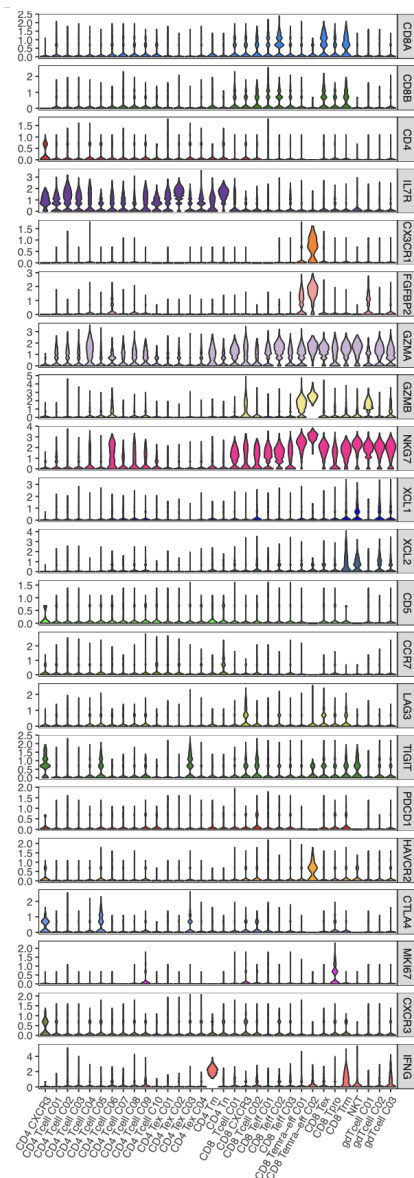**D**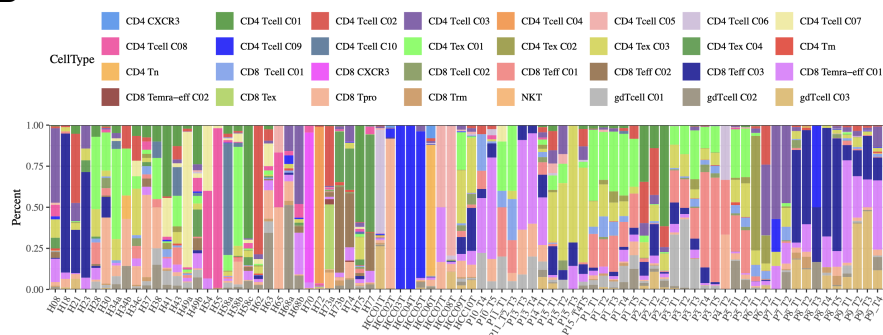**E**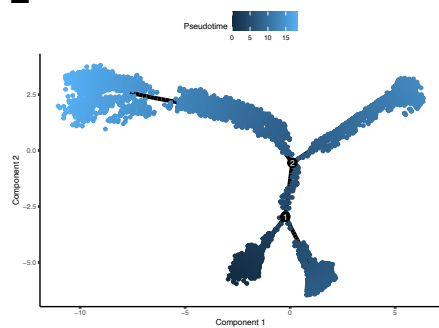**F**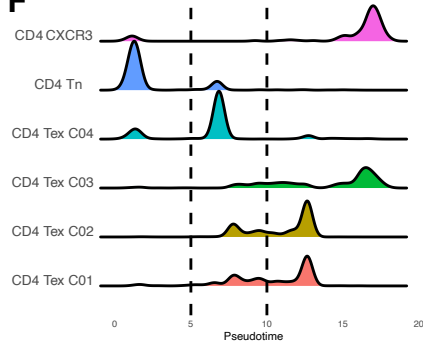**G**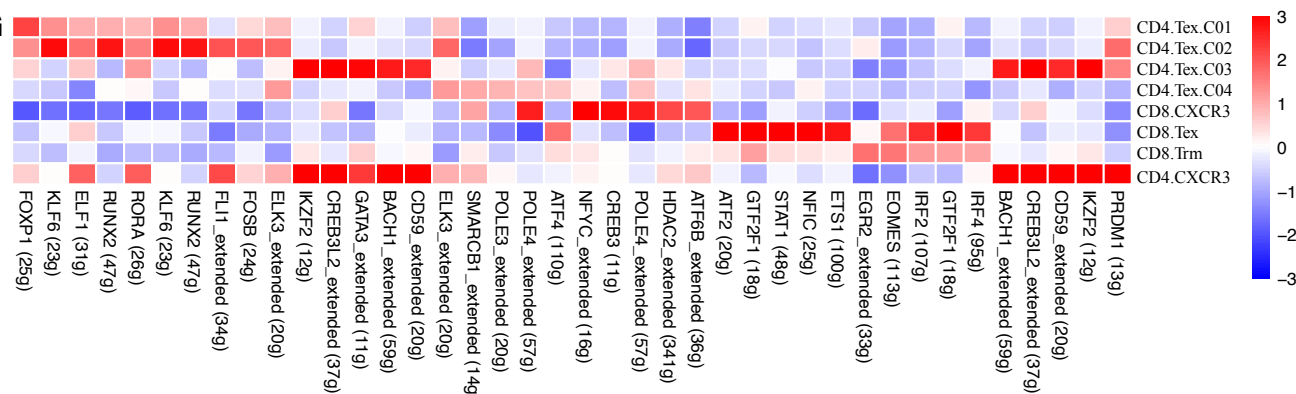

Supplement: Multimedia component 7 [file mmc7.pdf]

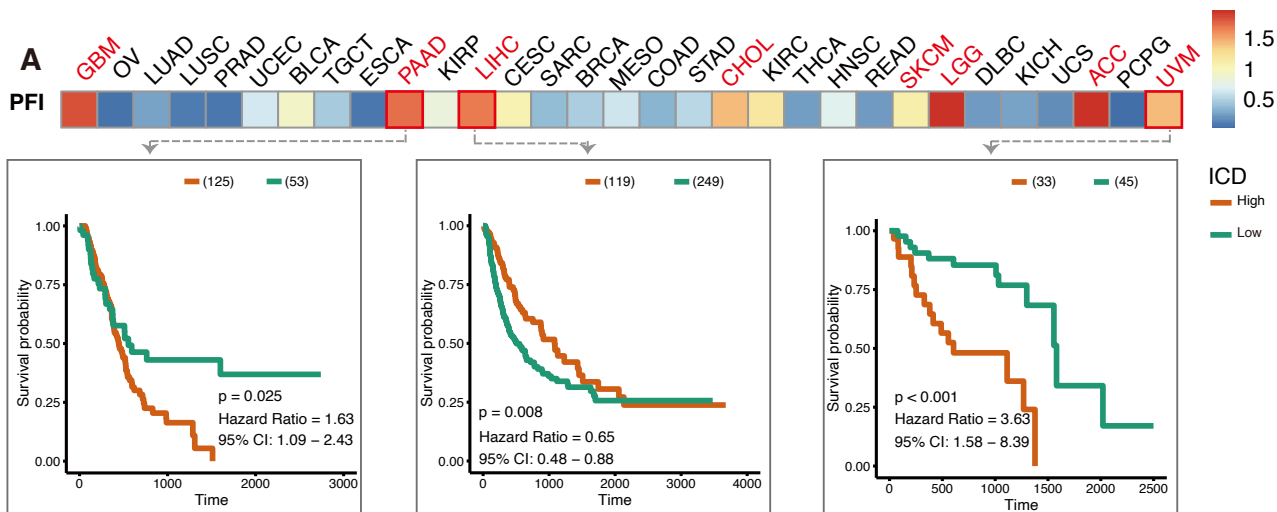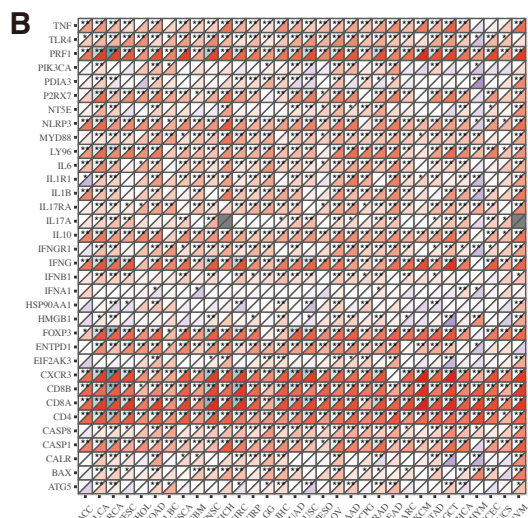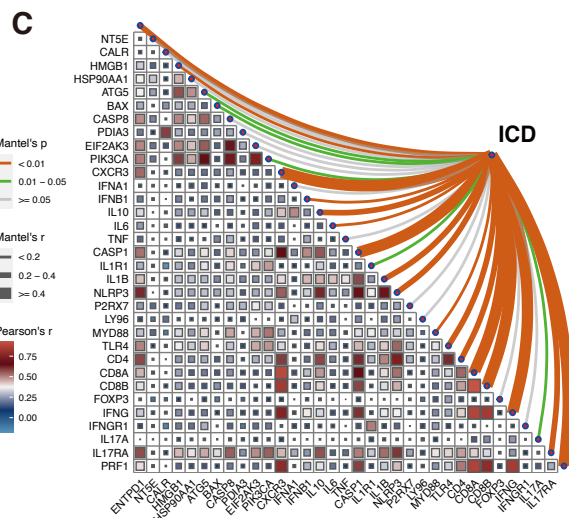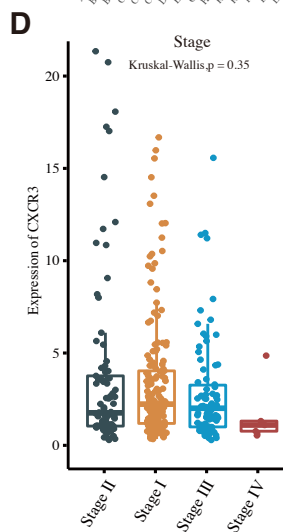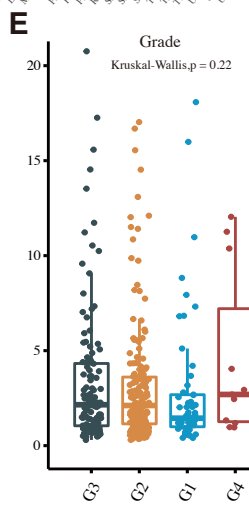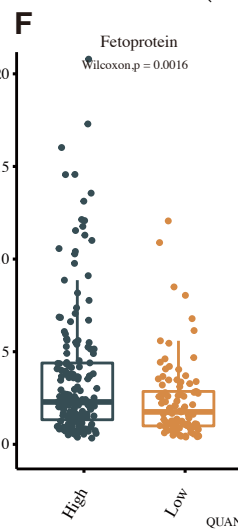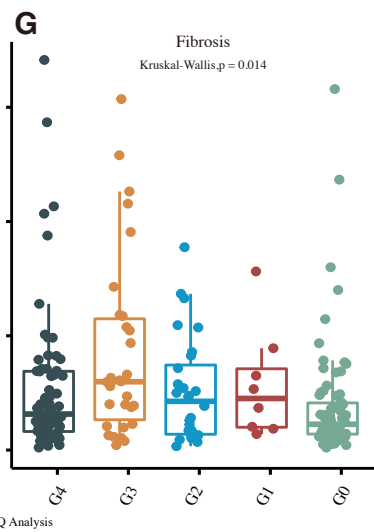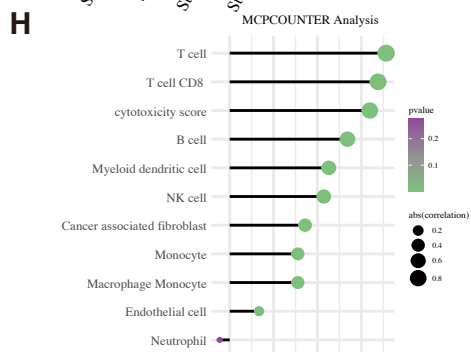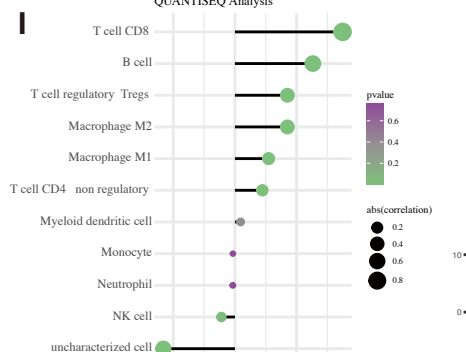

Supplement: Multimedia component 8 [file mmc8.pdf]

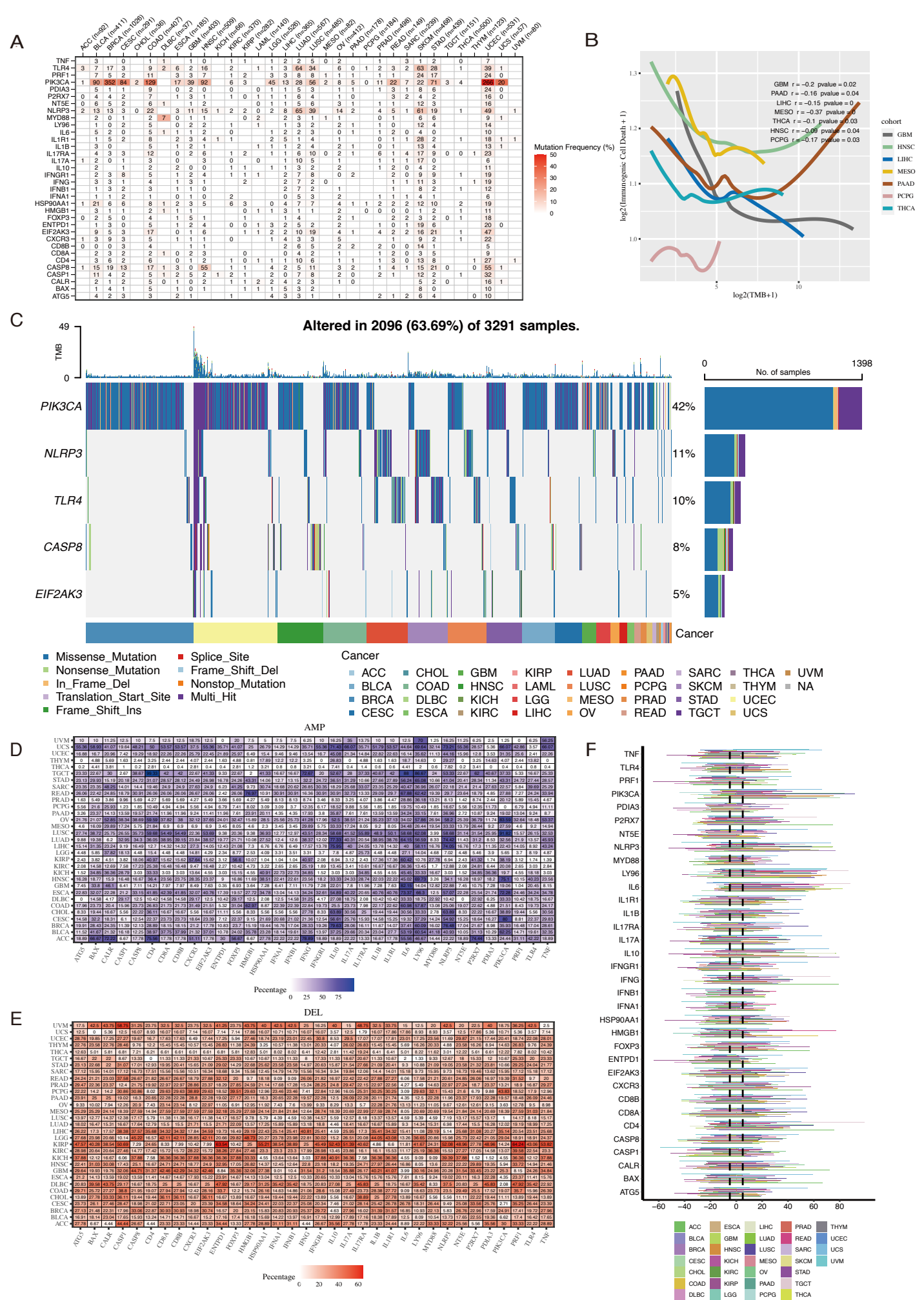

Supplement: Multimedia component 9 [file mmc9.pdf]

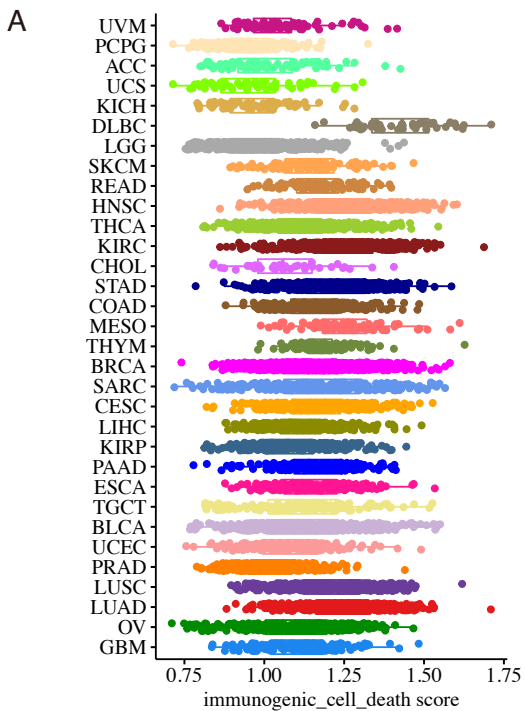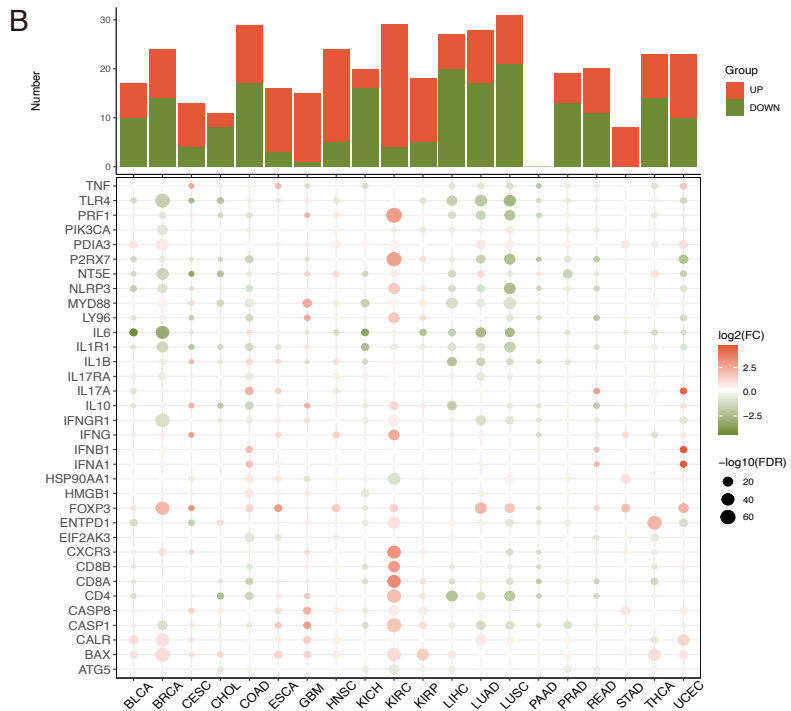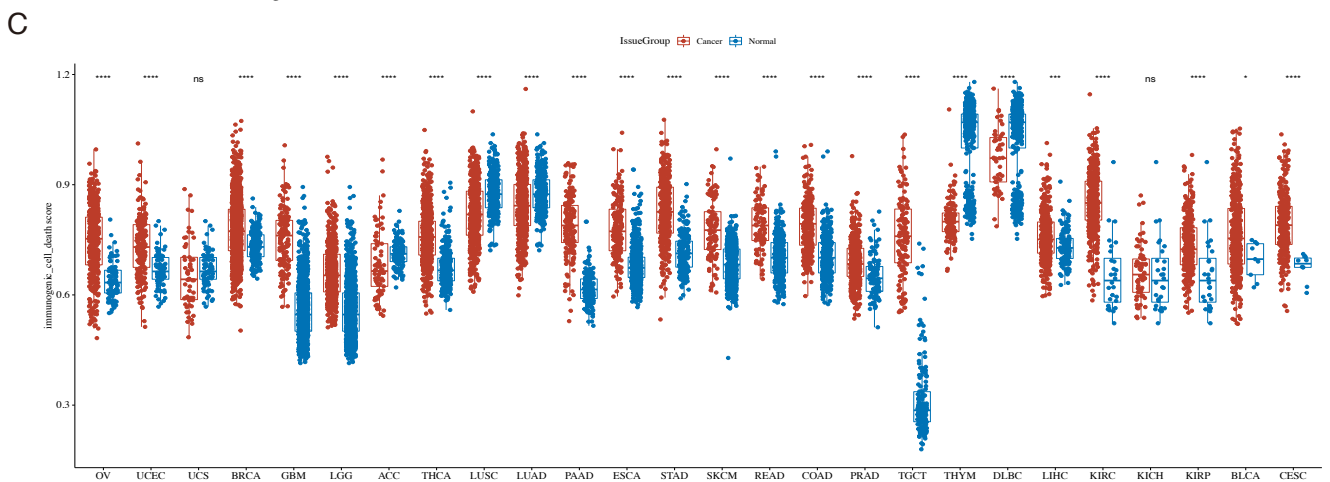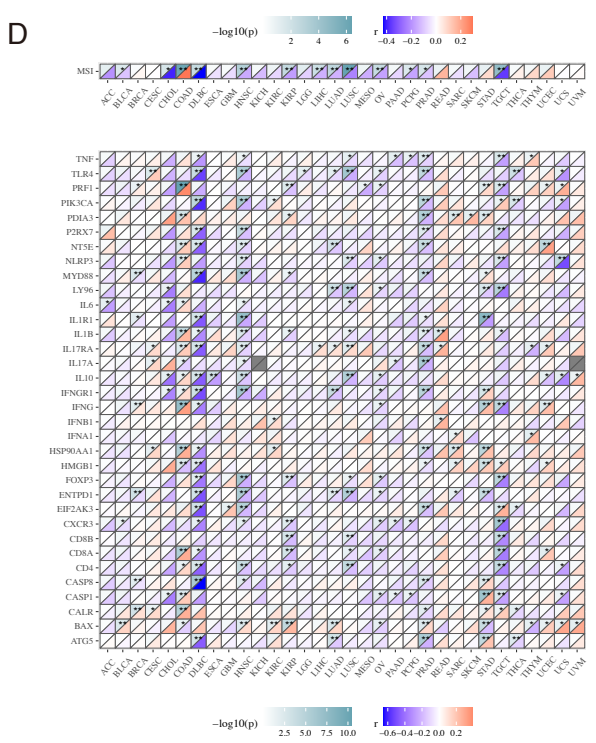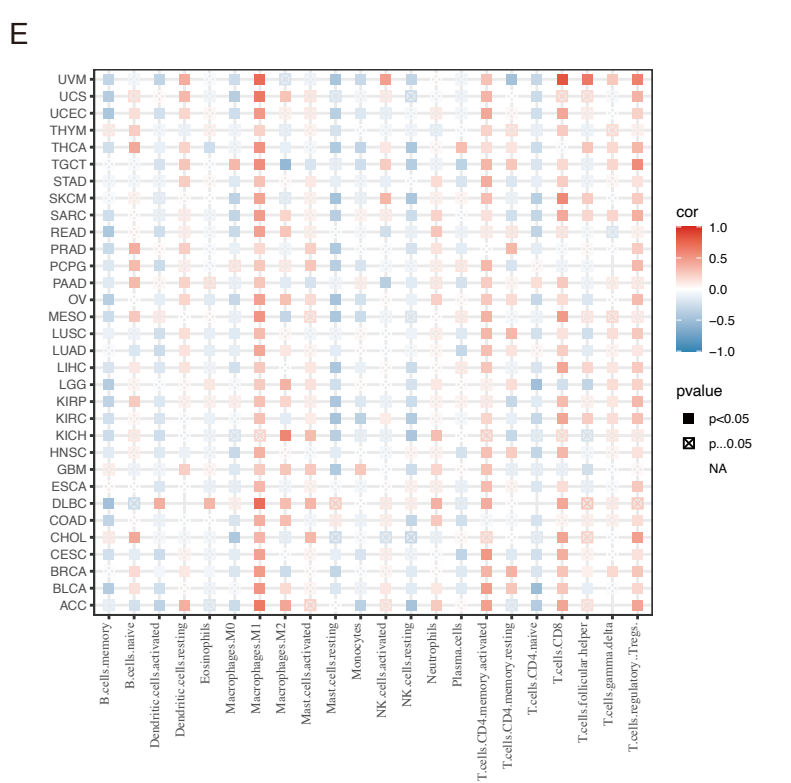

Supplement: Multimedia component 10 [file mmc10.pdf]
