# Supplementary material for: Pan-cancer immunogenic death analysis identifies key roles of CXCR3 and CCL18 in hepatocellular carcinoma
Source: Genes Dis. 2023 May 10;11(2):568–70. doi: 10.1016/j.gendis.2023.04.007 (PMC10491906; doi:10.1016/j.gendis.2023.04.007)
Supplement: Multimedia component 6 [file mmc6.pdf]

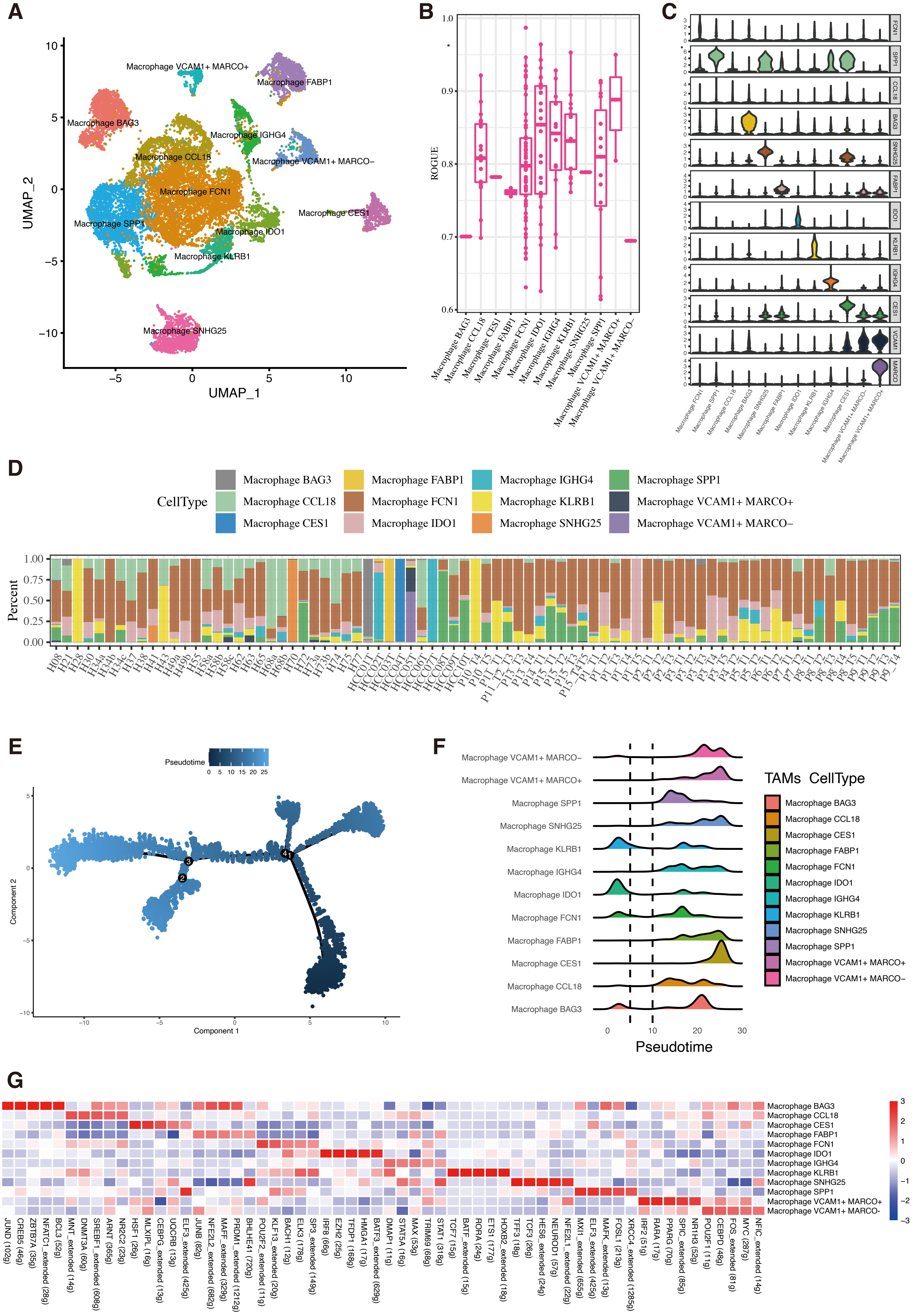
A
UMAP_1
UMAP_2
Macrophage VCAM1+ MARCO+
Macrophage FABP1
Macrophage BAG3
Macrophage IGHG4
Macrophage CCL18
Macrophage VCAM1+ MARCO−
Macrophage FCN1
Macrophage CES1
Macrophage SPP1
Macrophage IDO1
Macrophage KLRB1
Macrophage SNHG25
B
ROGUE
Macrophage BAG3
Macrophage CCL18
Macrophage CES1
Macrophage FABP1
Macrophage FCN1
Macrophage IDO1
Macrophage IGHG4
Macrophage KLRB1
Macrophage SNHG25
Macrophage SPP1
Macrophage VCAM1+ MARCO+
Macrophage VCAM1+ MARCO−
C
FCN1
SPP1
CCL18
BAG3
SNHG25
FABP1
IDO1
KLRB1
IGHG4
CES1
VCAM1
MARCO
D
CellType
Macrophage BAG3
Macrophage CCL18
Macrophage CES1
Macrophage FABP1
Macrophage FCN1
Macrophage IDO1
Macrophage IGHG4
Macrophage KLRB1
Macrophage SNHG25
Macrophage SPP1
Macrophage VCAM1+ MARCO+
Macrophage VCAM1+ MARCO−
Percent
E
Pseudotime
Component 1
Component 2
F
TAMs CellType
Pseudotime
G
JUND (102g)
CREB5 (46g)
ZBTB7A (35g)
NFATC1_extended (28g)
BCL3 (32g)
MNT_extended (14g)
DNMT3A (60g)
SREBF1_extended (608g)
ARNT (365g)
NR2C2 (23g)
HSF1 (26g)
MLXIPL (16g)
CEBPG_extended (13g)
UQCRB (13g)
ELF3_extended (425g)
JUNB (82g)
NFE2L2_extended (682g)
MAFF_extended (329g)
PRDM1_extended (1212g)
BHLHE41 (720g)
POU2F2_extended (11g)
KLF13_extended (20g)
BACH1 (12g)
ELK3 (178g)
SP3_extended (149g)
IRF8 (66g)
EZH2 (25g)
TFDP1 (168g)
HMGA1 (17g)
BATF3_extended (629g)
DMAP1 (11g)
STAT5A (16g)
MAX (53g)
TRIM69 (68g)
STAT1 (318g)
TCF7 (15g)
BATF_extended (15g)
RORA (24g)
ETS1 (177g)
HOXB2_extended (18g)
TFF3 (18g)
TCF3 (26g)
HES6_extended (24g)
NEUROD1 (57g)
NFE2L1_extended (22g)
MXI1_extended (655g)
ELF3_extended (425g)
MAFK_extended (13g)
FOSL1 (213g)
XRCC4_extended (1285g)
IRF2 (51g)
RARA (17g)
PPARG (70g)
SPIC_extended (85g)
NR1H3 (52g)
POU2F1 (11g)
CEBPD (48g)
FOS_extended (81g)
MYC (287g)
NFIC_extended (14g)
Macrophage BAG3
Macrophage CCL18
Macrophage CES1
Macrophage FABP1
Macrophage FCN1
Macrophage IDO1
Macrophage IGHG4
Macrophage KLRB1
Macrophage SNHG25
Macrophage SPP1
Macrophage VCAM1+ MARCO+
Macrophage VCAM1+ MARCO-
